# A New Synthetic Histone Deacetylase Inhibitor, MHY2256, Induces Apoptosis and Autophagy Cell Death in Endometrial Cancer Cells via p53 Acetylation

**DOI:** 10.3390/ijms19092743

**Published:** 2018-09-13

**Authors:** Umasankar De, Ji Yeon Son, Richa Sachan, Yu Jin Park, Dongwan Kang, Kyungsil Yoon, Byung Mu Lee, In Su Kim, Hyung Ryong Moon, Hyung Sik Kim

**Affiliations:** 1 School of Pharmacy, Sungkyunkwan University, 2066, Seobu-ro, Jangan-gu, Suwon 16419, Korea; umasankar9@gmail.com (U.D.); twiase@naver.com (J.Y.S.); richa.psit2009@gmail.com (R.S.); sow7826@naver.com (Y.J.P.); bmlee@skku.edu (B.M.L.); insukim@skku.edu (I.S.K.); 2 College of Pharmacy, Pusan National University, Busandaehak-ro 63 beon-gil 2, Geumjeong-gu, Busan 46241, Korea; 3607@pusan.ac.kr; 3 Comparative Biomedicine Research Branch, Division of Translational Science, National Cancer Center, 323 Ilsandong-gu, Goyang-si 10408, Korea; kyoon@ncc.re.kr

**Keywords:** histone deacetylase inhibitor, MHY2256, p53, apoptosis, autophagy, Ishikawa, endometrial cancer

## Abstract

We previously discovered a novel sirtuin (SIRT) inhibitor, MHY2256, that exerts anticancer activity through p53 acetylation in MCF-7 human breast cancer cells. We investigated the anticancer activity of MHY2256 against hormone-related cancer, an endometrial cancer with a poor prognosis. The IC_50_ values of MHY2256 were shown to be much lower than those of salermide, a well-known SIRT inhibitor. Furthermore, MHY2256 significantly reduced the protein expression and activities of SIRT1, 2, and 3, with similar effects to salermide. Particularly, MHY2256 markedly inhibited tumor growth in a tumor xenograft mouse model of Ishikawa cancer cells. During the experimental period, there was no significant change in the body weight of mice treated with MHY2256. A detailed analysis of the sensitization mechanisms of Ishikawa cells revealed that late apoptosis was largely increased by MHY2256. Additionally, MHY2256 increased G1 arrest and reduced the number of cell cyclic-related proteins, suggesting that apoptosis by MHY2256 was achieved by cellular arrest. Particularly, p21 was greatly increased by MHY225656, suggesting that cell cycle arrest by p21 is a major factor in MHY2256 sensitization in Ishikawa cells. We also detected a significant increase in acetylated p53, a target protein of SIRT1, in Ishikawa cells after MHY2256 treatment. In a mouse xenograft model, MHY2256 significantly reduced tumor growth and weight without apparent side effects. These results suggest that MHY2256 exerts its anticancer activity through p53 acetylation in endometrial cancer and can be used for targeting hormone-related cancers.

## 1. Introduction

Endometrial cancer is one of the most common gynecological malignancies, affecting approximately 142,000 women and causing an estimated 42,000 deaths worldwide [1,2]. Although the standard treatment for early-stage endometrial cancer is surgery, more advanced stages may require a multimodality treatment [3,4]. Therefore, new target therapies or therapeutic options are needed to decrease the morbidity and mortality rates observed for advanced stage endometrial cancer [5,6]. Recently, endometrial cancer was extensively studied at the molecular level to develop effective therapies using histone deacetylase (HDAC) inhibitors, which have shown their potential as therapeutic agents for endometrial cancer [7,8]. HDACs play an important role in regulating the epigenetic processes that lead to the expression of target genes in the development of multiple cancers [9,10].

**Figure 1:**
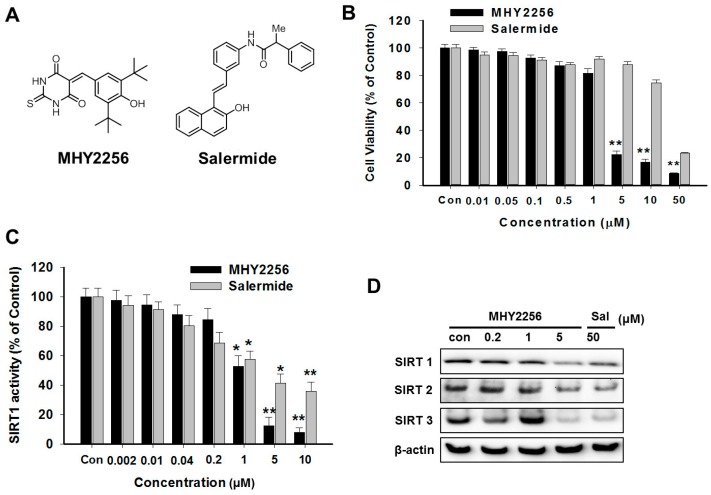
Cytotoxicity of MHY2256 on Ishikawa endometric cancer cells by reducing the sirtuin (SIRT) activity and protein levels. (**A**) The chemical structures of MHY2256 and salermide used in the present study. (**B**) The cells were treated with MHY2256 or salermide at various concentrations (0.1–50 μM) for 48 h. The cell viability was detected using a MTT (3-(4,5-dimethylthiazol-2-yl) -2,5-diphenyltetrazolium bromide) assay, and the data represent the mean ± standard error (SE) of three independent experiments. ** *p* < 0.01 indicate significant differences between the control and MHY2256. (**C**) The effects of MHY2256 and salermide on SIRT1 activity. The SIRT1 enzyme activity was measured using the SensoLyte^®^ 520 FRET SIRT1 assay kit. Statistical analysis was performed using one-way analysis of the variance, followed by Bonferroni’s multiple comparison tests. * *p* < 0.05 and ** *p* < 0.01 indicate significant differences between the control and treatment groups. (**D**) The effects of MHY2256 on different types of SIRT expression. The cells were treated with MHY2256 or salermide for 48 h, and then a Western blot analysis was performed.

Numerous HDAC inhibitors have been evaluated in clinical studies for the treatment of breast, cervical, and ovarian cancers, which are commonly correlated with hormone-dependent cancers in women. Furthermore, HDACs have a close relationship with signal transduction pathways and tumor suppression genes, which directly or indirectly alter cell proliferation in certain types of cancer cell lines [11,12,13]. Particularly, more specific targeting inhibitors for HDAC have focused on personalized medicine for treating the cancers affecting women [14,15]. A previous study demonstrated that the HDAC1, 2, 4, 6, or 7 series show strong immunoreactivity in undifferentiated endometrial sarcoma, and can be considered potential therapeutic targets [7]. Li et al. [11] found that a new HDAC inhibitor, FK228, significantly inhibited endometrial cancer cell proliferation and significantly induced apoptosis and cell cycle arrest at the G0/G1 phase in endometrial cancer cells. Moreover, FK228 treatment significantly increased the mRNA or protein expression of p53, p21, cleaved caspases-3, -7, or -8, and poly(ADP-ribose) polymerase (PARP). In our previous study, a novel synthesized class III HDAC inhibitor, MHY2256 (Figure 1A), reduced breast and ovarian cancer cell proliferation and induced apoptosis [16]. However, the exact role of class III HDAC sirtuin (SIRT) in p53 activation in endometrial cancer remains unclear.

In the present study, we synthesized the novel SIRT inhibitor, MHY2256, and investigated its anticancer activity against human endometrial cancer cells. Additionally, the anticancer potency of MHY2256 was compared to that of salermide, a selective SIRT inhibitor. To determine the anticancer activity of MHY2256 by SIRT inhibition, cell viability, the cell cycle regulation, and apoptosis- and autophagy-related molecule levels were measured.

## 2. Results

### 2.1. MHY2256 Is Highly Cytotoxicity to Ishikawa Endometrial Cancer Cells

The chemical structure of MHY2256 and salermide are shown in Figure 1A. Previously, we discovered that MHY2256 inhibits breast and ovarian cancer cell proliferation [16]. In this study, we tested whether MHY2256 also sensitizes endometrial cancer cells, another type of hormone-related cancer. We used the Ishikawa cancer cell line, which is a well-established endometrial cancer cell line. As shown in Figure 1B, MHY2256 significantly reduced the viability of the Ishikawa cells in a concentration-dependent manner. We compared the cytotoxicity using salermide, a well-known SIRT inhibitor. The measured IC_50_ value of MHY2256 against Ishikawa cells was 5.6 μM, which is approximately 10-fold lower than that of salermide. These results suggest that MHY2256 is highly cytotoxic towards endometric cancer cells. 

### 2.2. MHY2256 Reduces Both SIRT1 Enzyme Activity and SIRT Protein Levels in Ishikawa Cells

We measured the activity of the SIRT enzyme with our previous experimental protocol [16]. Salermide was used as a positive compound for the SIRT1 inhibitor. As shown in Figure 1C, MHY2256 significantly inhibited the activity of the SIRT1 enzyme, and the effect was totally dependent on the drug concentration. The IC_50_ of MHY2256 against the SIRT1 enzyme activity was 1.89 µM, which was lower than that of salermide (IC_50_, 4.8 µM). Next, the effect of MHY2256 on SIRT protein expression was examined by Western blot analysis. SIRT1, 2, and 3 levels were downregulated shown to be in the Ishikawa cancer cells following a high dose (5 µM) MHY2256 or salermide (50 µM) treatment (Figure 1D), suggesting that MHY2256 might target various SIRT proteins. Thus, MHY2256 exerts cytotoxic effects on endometric cancer cells by targeting SIRT proteins.

### 2.3. MHY2256 Inhibits Cell Cycle Distribution

**Figure 2:**
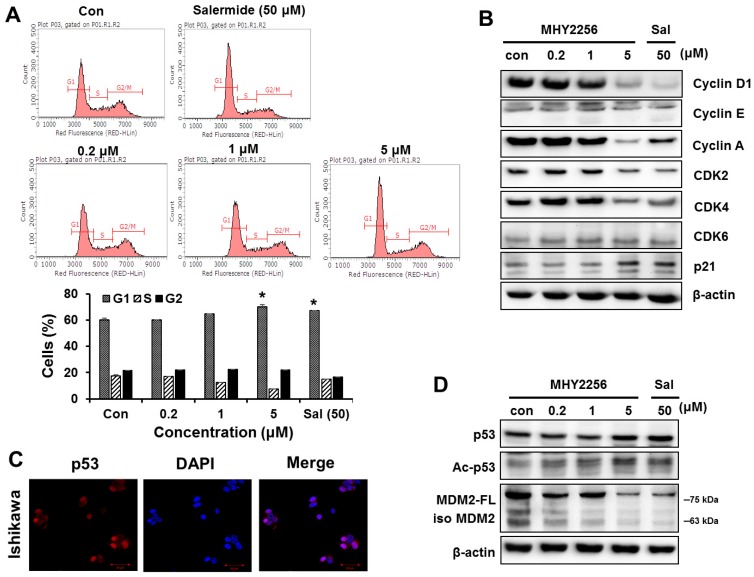
MHY2256 increases G1 arrest and reduces p53 levels via mouse double minute 2 (MDM2) degradation. (**A**) The Ishikawa cells were treated with the indicated concentrations for 48 h. The cells stained with propidium iodide (PI) were subjected to flow cytometric analysis in order to determine their distributions in each phase of the cell cycle. * *p* < 0.05 indicate significant differences between the control and treatment groups. (**B**) The effect of MHY2256 on the expression levels of cell cycle regulatory proteins. The cells were treated with MHY2256 (0.2, 1, or 5 μM) or salermide (50 μM) for 48 h, and then, protein levels were detected by Western blot analysis. Aliquots of proteins were immunoblotted with specific primary antibodies against cyclin D1, cyclin E, cyclin A, CDK2, CDK4, CDK6, and p21. (**C**) Basal expression levels of p53 protein in Ishigawa cancer cells. Immunofluorescence and fluorescence detection of p53 using rhodamine red-tagged secondary antibody were done using confocal microscopy (magnification ×400). (**D**) The effects of MHY2256 on expression of p53, acetylated p53 (Ac-p53), full length of MDM2 (MDM2-FL), and MDM2 isomers (Iso MDM2). The Ishikawa cells were treated with MHY2256 and salermide for 48 h, and then Western blot analysis was performed.

Data from earlier experiments showed that the SIRT inhibitors achieve their anticancer activity through cell cycle arrest, which is completely dependent on the inhibitors’ conditions [17,18]. We examined the effect of MHY2256 on cell cycle distribution by flow cytometry. The cells were treated with the indicated concentrations of MHY2256 (0.2, 1 or 5 μM) or salermide (50 μM) for 48 h. MHY2256 markedly increased the number of Ishikawa cells at the G1 phase and decreased S phase (Figure 2A). MHY2256-mediated cell cycle distribution was similar to that of salermide, suggesting that the SIRT1 inhibitor arrests the G1 phase of Ishikawa cells. The effect of MHY2256 on the expression levels of the cell cycle-related proteins was confirmed by Western blot analysis. MHY2256 markedly reduced the cyclin and cyclin-dependent kinase (CDK) protein levels, indicating that these molecules are associated with the G1 phase cell cycle checkpoints (Figure 2B). Additionally, MHY2256 significantly increased the expression of p21, suggesting that MHY2256 arrests the cell cycle mainly through p21 upregulation.

### 2.4. MHY2256 the Steady State Level of p53 Protein in Increase via Downregulation of the MDM2 Expression

It has been reported that the SIRT inhibitors upregulate p53 in various cancer cells [16,19]. Therefore, we investigated whether MHY2256 sensitizes the Ishikawa cells through p53 regulation. As shown in Figure 2C, a high basal expression level of p53 was shown in the Ishikawa cells. MHY2256 markedly increased acetylated and total p53 levels. Moreover, MHY2256 markedly reduced the expression of mouse double minute 2 (MDM2), an important negative regulator of p53 (Figure 2D). The changes in the MHY2256-mediated p53 signaling pathway were similar to those caused by salermide, suggesting that the SIRT1 inhibitors activate p53 by degrading MDM2. This suggests that the blocking of MDM2-p53 binding through the increasing of p53 acetylation via MHY2256 can serve as cytotoxicity mechanism in Ishikawa endometrial cancer cells. This led us to investigate whether Ishikawa cells subsequently induced apoptosis because of the p53 activation.

### 2.5. MHY2256 Induces Apoptotic Cellular Death in Ishikawa Cells

**Figure 3:**
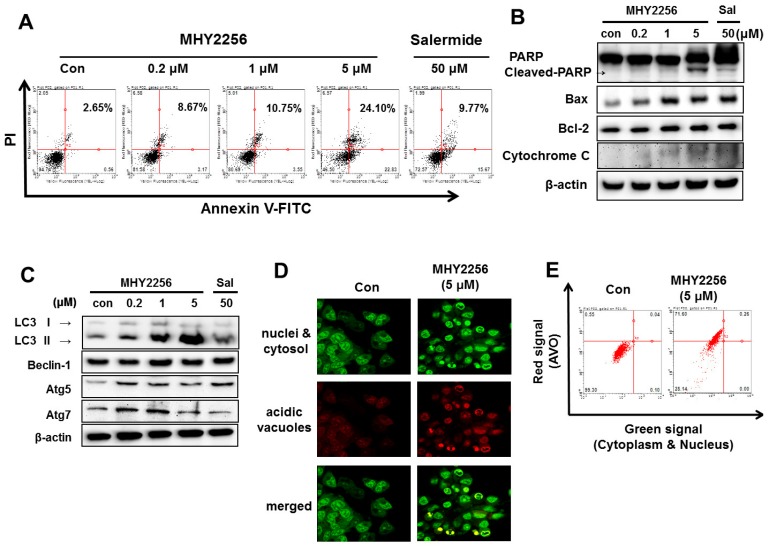
MHY2256 induced apoptosis and autophagy in Ishikawa endometric cancer cells. (**A**) The cells were treated with MHY2256 (0.2, 1, 5 µM) or salermide (50 µM) for 48 h. Apoptotic cell death was measured by Annexin V-FITC and propium iodide (PI) double staining using flow cytometry. Scatter plots indicate the percentages of viable cells, necrotic cells, and early or late apoptotic cells. (**B**) To determine the apoptosis-related protein levels, Western blot analysis was performed following each experiment. The protein levels were normalized by comparison with β-actin levels. (**C**) To evaluate the MHY2256-induced autophagy, Western blot analysis was performed to detect the LC3-I/II, Beclin-1, Atg5, and Atg7 protein expressions. (**D**) Detection of acidic vesicular organelles (AVOs) in Ishikawa cells. The cells were treated with MHY2256 (5 µM) for 48 h before stained with 1 µg/mL acridine orange for 15 min. Cell were examined by fluorescence microscopy. Representative images of cells from three independent experiments were shown (magnification ×400). (**E**) AVOs were analyzed by flow cytometry. Representative histograms of acridine orange-stained cells after treated with MHY2256 for 48 h, displaying the x and y axes as increasing green and red fluorescence intensities, respectively, are shown.

Annexin V-fluorescein isothiocyante/propidium iodide (FITCT/PI) staining was performed to assess the MHY2265-mediated early and late apoptosis cell death in the Ishikawa cells. Our results showed that the number of MHY2256-induced early and late stage apoptotic cells significantly increased in a concentration-dependent manner (Figure 3A). To identify the detailed mechanism behind the MHY2256 mediated apoptosis cell death, we measured the apoptosis-related protein using Western blot analysis. Our experimental data demonstrated that MHY2265 upregulates the expression of cleaved PARP (Figure 3B). Increased cytochrome c release was also detected following MHY2256 treatment (Figure 3B). These results confirm that MHY2256 sensitizes Ishikawa cancer cells to apoptosis. Furthermore, Bax expression significantly increased in a concentration-dependent manner, however, Bcl-2 expression was slightly increased by either MHY2256 or salermide treatment (Figure 3B). MHY2256 has a large effect on late apoptosis, suggesting that apoptotic cellular death by MHY2256 is highly cytotoxic, even at low doses.

### 2.6. MHY2256 Induces Autophagic Cell Death

We previously showed that MHY2256 significantly increases the autophagic cell death of breast cancer cells [16]. To assess whether this also occurs for Ishikawa cells, Western blot analysis, and acridine orange staining were performed. As shown in Figure 3C, low concentrations of MHY2256 (0.2 and 1 µM) markedly increased the levels of LC3-II and autophagy-related gene 5 (ATG5). In contrast, salermide did not greatly increase LC3-II (Figure 3C), suggesting that MHY2256 is a novel SIRT inhibitor which acts differently to the well-known SIRT inhibitor, salermide, further confirming the autophagy vacuoles, as shown through acridine orange staining. MHY2256 significantly increased the red florescence acidic vesicular organelles (AVOs) in the Ishikawa cell lines at 48 h (Figure 3D). A flow cytometric analysis after the acridine orange staining also showed an increased red fluorescence intensity following the drug treatment. Histogram profiles were drawn to show the mean fluorescence intensity of the control and drug-treated cells (Figure 3E). The increased autophagy in Ishikawa cells contributes to highly apoptotic cytotoxicity of MHY2256.

### 2.7. MHY2256 Inhibits Ishikawa Endometric Cell Tumors in a Xenograft Model

**Figure 4:**
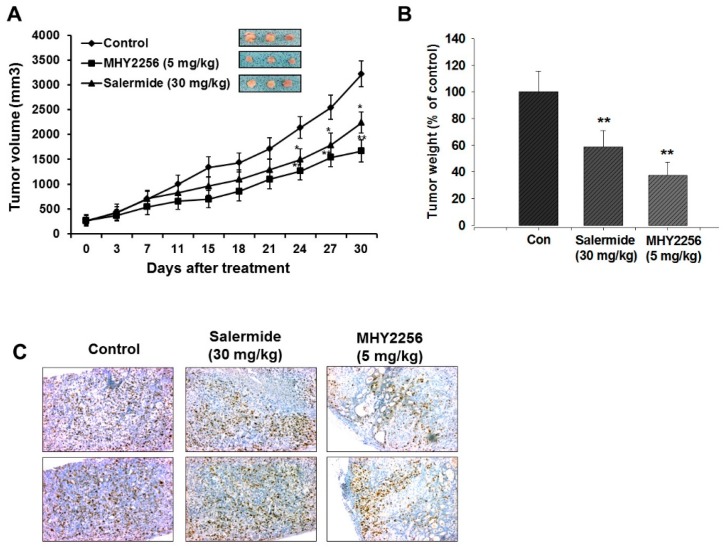
MHY2256 significantly reduces the growth of Ishikawa endometrial cancer cells in nude mice. Mice with pre-established tumors were randomized into three groups, with five mice in each group. The vehicle control, MHY2256 (5 mg/kg, twice/week, intrperitoneal (i.p.) injection), and salermide (30 mg/kg/week, i.p.) were administered to the tumor-bearing mice. (**A**) The mean tumor volumes during the 30-day treatment for each treatment group. (**B**) Each bar represents the inhibition rate (% of control) of the mean tumor weight. (**C**) The tumors were fixed in 10% formalin and embedded in paraffin. Immunohistochemical staining for Ki-67 was measured in the tumors. Magnification = ×200. Scale bar = 50 μM. The representative images were recorded under a 40× objective lens. In all graphs, the results are presented as the mean ± SE per group. Statistical analysis was performed using one-way analysis of the variance (ANOVA), followed by Bonferroni’s multiple comparison test. * *p* < 0.05 and ** *p* < 0.01 indicate significant differences between the control and treatment groups.

To evaluate the anticancer effects of MHY2256 in an in vivo xenograft model, nude mice were inoculated with Ishikawa cells and treated with MHY2256 (5 mg/kg) or salermide (30 mg/kg) for four weeks. In the MHY2256 treatment group, tumor volumes and weights were significantly reduced by 60%, relative to the control group (Figure 4A,B). Similarly, salermide time-dependently inhibited tumor growth (Figure 4A,B). No significant adverse effects or body weight changes were observed following the MHY2256 treatment. Taken together, these data demonstrate that the antitumor activity of MHY2256 towards endometrial tumor cell proliferation is related to reduce proliferating cell nuclear antigen expression in tumor tissues (Figure 4C).

## 3. Discussion

SIRT has shown promising anticancer effects, but has not been widely examined in clinical trials. Previously, we investigated the mechanisms of the SIRT inhibitor, sirtinol, which promotes cell death in the MCF-7 cell line through the induction of autophagy [20]. Moreover, we screened and identified a novel synthesized SIRT inhibitor, MHY2256, which is highly cytotoxic towards MCF-7 and SKOV-3 cancer cells [16]. In the current study, we evaluated the anticancer activity of MHY2256 in endometrial cancer. 

Firstly, we demonstrated that MHY2256 significantly reduces cell proliferation in the Ishikawa cell line by directly inhibiting the SIRT protein levels and activity. Thus, MHY2256 can greatly inhibit female-related cancers by inhibiting SIRT protein levels. Importantly, the anti-tumor effects of MHY2256 were demonstrated in an in vivo xenograft model, in which Ishikawa tumor growth was significantly retarded by MHY2256 injection. Our in vivo results revealed no side effects in animals injected with MHY2256, suggesting that MHY2256 can be applied in endometrial cancer, in combination with other treatments or as a single treatment. In order to determine the molecular mechanisms of MHY2256 against endometrial cancer cell proliferation, we first measured the apoptotic cell death pathway underlying the cytotoxic effect of MHY2256. The expression levels of apoptosis-related proteins were altered by MHY2256 treatment. Interestingly, cytotoxic effects of low-dose MHY2256 were observed during the late-stage apoptosis of Ishikawa cancer cells, suggesting that MHY2256 is selectively cytotoxic towards endometrial cancer cells. Therefore, our data indicate that this inhibitor can be used for targeted cancer therapeutics to treat endometrial carcinoma. 

Previous studies have shown that the SIRT inhibitors induce apoptosis through several mechanisms in a variety of cancer cells [21,22]. Furthermore, SIRT inhibitor administration activates the proapoptotic protein, an upstream mediator of mitochondrial membrane disruption, through the overexpression of antiapoptotic Bcl-2, which is known to be downregulated by SIRT expression [23,24]. Previous studies have indicated that modifications to the histone proteins are associated with the tumorigenesis of endometrial cancer development [25,26,27]. However, a dual function of SIRT1 in tumor promotion and suppression has been described in different cancer types [28,29]. SIRT7 functions as an oncogene and is upregulated in many cancer types [30]. However, no link has been shown between SIRT1 and 7 [31]. Therefore, different types of SIRTs play vital roles in endometrial cancer through to their expression, with similar effects found in other cancer types. 

Our previous study showed that MHY2256 significantly increases autophagic cell death in female-related cancer cells [16]. We also tested whether autophagic apoptosis increases following MHY2256 treatment. We found that increased autophagy in Ishikawa cells were due to the high apoptotic cytotoxicity of MHY2256. Thus, MHY2256 may use conserved autophagic death mechanisms to induce highly cytotoxic apoptosis in female-related cancers. We found that p21 was greatly increased by MHY2256. Thus, p21 may be a key target for the previously-shown G1 phase arrest by MHY2256, and may also contribute to the increased late apoptosis caused by MHY2256. It has been reported that SIRT inhibitors upregulate p53 in some cancers [15,17,18]. We also found that the blocking of MDM2-p53 binding through p53 deacetylation inhibition via MHY2256 can serve as a mechanism to sensitize Ishikawa endometric cancer cells. MHY2256 shows the potential for the development of drugs to target p53. Lara et al. [22] reported that the SIRT1-dependent proapoptotic activity of salermide is not associated with p53. Salermide mediates apoptotic cell death; this is dependent on the activation of proapoptotic genes that are directly or indirectly suppressed by SIRT1 in cancer cells. Furthermore, in MCF-7 cells, it was shown that the antitumor qualities of sirtinol and salermide are produced through an increasing the acetylation form of SIRT1/2, which altered the tubulin and p53 expression in an animal model. [18,20]. In this study, we found that MHY2256 profoundly inhibits endometrial cancer cell proliferation, and induces apoptosis that causes a change in the cell morphology. Overall, our experimental data suggest that MHY2256 acts as a novel SIRT inhibitor, which more categorically targets both SIRT1 and SIRT2 to induce cell death and p53 acetylation. Furthermore, the acetylation of p53 following SIRT inhibition may cause a reduction in p53 degradation as well as accelerating the p53 activity [22,32]. For further confirmation, additional studies are needed to verify the MHY2256 mediated anticancer effect in endometrial cancer cells. In summary, based on the previously-shown in vitro anticancer response of MHY2256 on endometrial cancer cells, antitumor activity was evaluated in a tumor xenograft model using nude mice. MHY2256 inhibited tumor growth by 53% in the Ishikawa cell xenografts compared to those treated with the vehicle control alone. These results suggest that MHY2256 not only inhibits Ishikawa cell growth in vitro, but also greatly inhibits endometrial tumor cell growth in vivo. The inhibitory effects of MHY2256 were also evaluated with the proliferative index Ki67, one of the key markers for cell proliferation. We found that the MHY2256 markedly reduced the expression of Ki67-positive cells compared to the control group. Thus, we conclude that MHY2256 significantly inhibits cell proliferation as well as tumor growth in both in vitro and in vivo studies of endometrial cancer cells.

## 4. Materials and Methods

### 4.1. Chemistry

Whole, commercially-purchased reagents were used directly, without purification. Mass spectrometry (MS) data were collected from an Expression CMS (Advion, Ithaca, NY, USA). CDCl_3_ and dimethyl sulfoxide (DMSO)-*d*_6_ were used for nuclear magnetic resonance (NMR) and chemical transformation unit and are expressed as parts per million (ppm), with an altering residual solvent or deuterated peaks (*δ*_H_ 7.24 and *δ*_C_ 77.0 for CDCl_3_, *δ*_H_ 2.50 and *δ*_C_ 39.7 for DMSO-*d*_6_). For the detection of NMR data, the Unity INOVA 400 spectrometer or a Varian Unity AS500 spectrometer (Agilent Technologies, Santa Clara, CA, USA) was used. The coupling constant is expressed in Hz. All of the reactions were controlled by thin-layer chromatography atmospheric nitrogen using Merck precoated 60F_254_ plates (Billerica, MA, USA).

### 4.2. Synthesis of MHY2256

A solution of 2,6-di-*tert*-butylphenol (10 g and 48.47 mmol) and hexamethylenetetramine (6.79 g and 48.43 mmol) in acetic acid (20 mL) and water (4 mL) was refluxed for 7 h. After cooling, the reaction mixture was filtered and washed with methyl alcohol and water to give pure 3,5-di-*tert*-butyl-4-hydroxybenzaldehyde (10.40 g and 91.6%) as a solid. ^1^H NMR (500 MHz, CDCl_3_) *δ* 9.85 (s, 1 H, CHO), 7.73 (s, 2 H, 2-H, 6-H), 5.85 (s, 1 H, OH), 1.48 (s, 18 H, 2 × *tert*-C_4_H_9_); ^13^C NMR (100 MHz, CDCl_3_) 192.0 (*C*HO), 159.9 (4C), 136.7 (3C, 5C), 129.0 (1C), 127.9 (2C, 6C), 34.6 (2 × *C*[CH_3_]_3_), 30.3 (2 × C[*C*H_3_]_3_). To a stirred solution of 3,5-di-*tert*-butyl-4-hydroxybenzaldehyde (300 mg, 1.28 mmol) in ethanol (12 mL) and water (12 mL), 2-thioxodihydropyrimidine-4,6(1*H*,5*H*)-dione (thiobarbiturate, 185 mg, 1.28 mmol) was added, and the reaction mixture was refluxed for 16 h. After evaporating the ethanol, the mixture was filtered and washed with water and methylene chloride to give pure 5-(3,5-di-*tert*-butyl-4-hydroxybenzylidene)-2-thioxodihydropyrimidine- 4,6(1*H*,5*H*)-dione (MHY2256, 378.2 mg, 82%) as a solid. ^1^H NMR (400 MHz, DMSO-*d*_6_), 12.21 (s, 2 H, 2 × NH), 8.31 (s, 2 H, 2-H, 6-H), 8.20 (s, 1 H, vinylic H), 1.36 (s, 18 H, 2 × *tert*-C_4_H_9_); LMHR(ESI−) 359 (M − H).

### 4.3. Cell Culture

The Ishikawa cancer cells were kindly provided by Jacques Simard (CHUL Research Center, Quebec, QC, Canada). The cells were maintained as monolayers at 37 °C in Dulbecco Modified Eagle Medium (DMEM) (Gibco, Grand Island, NY, USA) containing 10% heat-inactivated fetal bovine serum (Gibco) and 1% penicillin/streptomycin (Gibco), in an atmosphere containing 5% CO_2_/air.

### 4.4. SIRT1 Activity Assay

To determine the SIRT1 activity, we used a SensoLyte^®^ 520 fluorimetric SIRT1 activity assay kit (AnaSpec, Fremont, CA, USA), and the SIRT1 activity was measured with the SensoLyte^®^ 520 fluorimetric SIRT1 activity assay kit. In accordance with the manufacturer’s protocol, different concentrations of the drug and vehicle were incubated with SIRT1 enzymes at 37 °C in the presence of an SIRT1 fluorimetric VICTOR X2 (Perkin Elmer, Waltham, MA, USA), with excitation at 490 nm and emission at 520 nm. The fluorescence was measured using experimental data and calculated using GraphPad Prism7 (GraphPad Software, San Diego, CA, USA).

### 4.5. Cytotoxicity Assay

3-(4,5-Dimethylthiazol-2-yl)-2,5-diphenyltetrazolium bromide (MTT; 5 mg/mL, Sigma, St. Louis, MO, USA) was used to measure the cytotoxicity of MHY2256 in the Ishikawa cells. The cells were seeded in 96-well plates with 3 × 10^3^ cells per well. After 24 h, the cells were incubated with various concentrations of MHY2256 and salermide for 48 h. The treated cells were further incubated with MTT reagent for 4 h at 37 °C in the dark. Later, the formed formazan crystals were dissolved in 100 µL of DMSO at 37 °C for 10 min. The data was measured in a VERSA Max Microplate Reader (Molecular Devices, Sunnyvale, CA, USA) at 540 nm. The half-maximal inhibitory concentration (IC_50_) values were calculated using SigmaPlot 10.0 software (Systat Software, Chicago, IL, USA).

### 4.6. Western Blot Analysis

The Ishikawa cells were treated with MHY2256 (0.2, 1, and 5 μM) or salermide (50 μM). After 48 h, the treated cells were collected and washed with cold Dulbecco’s phosphate-buffered saline (DPBS). Later, the cells were suspended in a PRO-PREP^TM^ protein extract solution (iNtRON, Seongnam, Korea) for 15 min. The total protein isolate and the protein concentration were measure with a protein assay kit (Bio-Rad, Hercules, CA, USA). The same amount of protein was loaded on a 6–15% sodium dodecyl polyacrylamide (PAGE) gel. After electrophoresis, the proteins were transferred to polyvinylidene difluoride membranes (Millipore, Billerica, MA, USA), and the membranes were incubated with a TNA (50 mM Tris-HCl, pH 8; 100 mM NaCl, and 0.4 M l-arginine) buffer containing 5% skim milk for blocking for up to 1 h. Then, the membranes were incubated with different kinds of primary antibodies at 4 °C overnight. The following day, after washing the membrane with a TNA buffer for 1 h, it was incubated with a secondary antibody, horseradish peroxidase-conjugated anti-mouse or anti-rabbit antibody (1:10,000, Santa Cruz Biotechnology, Dallas, TX, USA), for 1 h at room temperature, and was then further washed for 1 h. Finally, the membranes were developed with an enhanced chemiluminescence (ECL)-plus kit (Amersham Biosciences, Amersham, UK).

### 4.7. Flow Cytometry Analysis

The cells were treated with various concentrations of MHY2256 (0.2, 1, and 5 μM) or salermide (50 μM) for 48 h. Both the suspended and attached cells were collected and washed with (DPBS). Then, all of the cells were washed with 1% bovine serum albumin (BSA) and were then fixed in 95% ice-cold ethanol, which contained 0.5% tween 20, for 2 h at −20 °C. After fixation, an equal number of cells (1 × 10^6^) were washed with cold 1% BSA and cell stained with propidium iodine (PI, 10 μg/mL and 100 μg/mL RNase in phosphate-buffered saline (PBS)). Then, they were incubated for 30 min at room temperature and protected from light. The cells were analyzed using a flow cytometry system (BD Biosciences, San Jose, CA, USA).

### 4.8. Annexin V-FITC/PI Binding Assay

The cells were treated with MHY2256 (0.2, 1, or 5 μM) or salermide (50 μM) for 48 h. The same number of cells was collected and washed twice with cold PBS. All cells were suspended in a 100 µL binding buffer at a density of 1 × 10^5^ cells/mL. After that, 5 µL of FITC-conjugated annexin V and 5 µL of PI were added for 15 min at room temperature. After that, the remaining 400 µL of binding buffer was mixed gently to avoid a vortex, and the cells were directly analyzed by fluorescence-activated cell sorting (BD Biosciences).

### 4.9. Detection of Acidic Vesicular Organelles (AVOs)

The cells (1 × 10^5^) were seeded in T-75 flasks followed by treatment with control (0.1% DMSO) and MHY2256 (5 μM) for 48 h. Cells were stained with acridine orange (1 µg/mL) for 15 min, washed with DPBS, and fixed using 100% methanol followed by examining under a fluorescence microscope. AVOs were also detected using flow cytometry after the cell pellet was stained with acridine orange for 20 min followed by washing the cells with DPBS. Green (510–530 nm) and red (650 nm) fluorescence emission from Ishikawa cells (1 × 10^4^) illuminated with blue (488 nm) excitation light was measured with a Guava EasyCyte Plus Flow Cytometer (Merck Millipore, Billerica, MA, USA).

### 4.10. In Vivo Tumor Xenograft Model

An in vivo study was performed using six-week-old female nude mice (BALB-c nu/nu, Japan SLC, Inc., Hamamatsu, Shizuoka, Japan) with temperature control (22 ± 2 °C) and light control (12 h light/dark cycle) in filtered-air laminar-flow cabinets. The Sungkyunkwan University Animal Care Committee approved (protocol number SKKU-2018-048-07, 19 April 2018) the experimental procedure. The Ishikawa cells (2 × 10^7^) were collected, and 0.1 mL of a serum-free medium and 0.1 mL of Metrigel (BD Biosciences, 35434, maintain the ratio 1:1) were injected subcutaneously (s.c.). Before the drug administration, the tumor sizes were checked regularly until they reached 200 mm^3^, and they were then randomized into three (*n* = 5) groups. MHY2256 (5 mg/kg) or salermide (30 mg/kg) were injected intraperitoneally (twice per weekly) into the mice for 30 days, and 0.1% DMSO was used in the same manner for the control. The size of the tumors was measured with calipers, and the tumor volumes were calculated with the standard formula of width^2^ × length × 0.52. After the final drug administration, all of the nude mice were sacrificed. Images of the detached tumor group were taken, and some parts of the tumors were frozen in liquid nitrogen and stored at −80 °C, and the remaining parts were fixed in 10% formalin for further analysis. A formalin fixation tumor sample was embedded in paraffin, and then, Ki-67 expression was measured using immunohistochemical staining. The histological images were observed under 40 × magnification.

### 4.11. Statistical Methods

The experimental data were performed at least three times, and are expressed as the mean ± standard error (SE). For the determination of statistically significance, we used a one-way analysis of variance (ANOVA) and then compared it with Bonferroni’s multiple comparison tests. * *p* < 0.05 and ** *p* < 0.01 indicate the significant differences between the control and the treatment groups. All of the statistical comparisons were performed using SigmaPlot graphing software and the Statistical Package for the Social Sciences v.13 (SPSS, Inc., Chicago, IL, USA).

## 5. Conclusions

Our results reveal the anticancer mechanism of a new HDAC inhibitor in regulating tumor suppressor genes, and consequently, its potential therapeutic role for endometrial cancer. In this study, the SIRT inhibitor, MHY2256, induced anticancer activities through multiple cell death mechanisms, such as apoptosis, autophagy, and G1 phase cell cycle arrest in Ishikawa cells. Furthermore, MHY2256 markedly increased the levels of acetylated p53 and, at the same time, reduced MDM2 expression. Therefore, MHY2256 is a potential SIRT-targeted agent against female-related cancers, particularly in patients with high p53 expression. Additional studies are needed to evaluate the female-specific effects of SIRT inhibitors in cancer.

## References

[1] Amant F., Moerman P., Neven P., Timmerman D., Van Limbergen E., Vergote I. (2005). Endometrial cancer. Lancet.

[2] Tran A.Q., Gehrig P. (2017). Recent Advances in Endometrial Cancer. F1000Research.

[3] Arend R.C., Jones B.A., Martinez A., Goodfellow P. (2018). Endometrial cancer: Molecular markers and management of advanced stage disease. Gynecol. Oncol..

[4] Matias-Guiu X., Prat J. (2013). Molecular pathology of endometrial carcinoma. Histopathology.

[5] DeAngelis J.T., Farrington W.J., Tollefsbol T.O. (2008). An overview of epigenetic assays. Mol. Biotechnol..

[6] Damaskos C., Garmpi A., Spartalis E., Kalampokas E., Kalampokas T., Margonis G.A., Schizas D., Andreatos N., Angelou A., Lavaris A. (2018). Targeting histone deacetylases in endometrial cancer: A paradigm-shifting therapeutic strategy?. Eur. Rev. Med. Pharmacol. Sci..

[7] Baek M.H., Park J.Y., Rhim C.C., Kim J.H., Park Y., Kim K.R., Nam J.H. (2017). Investigation of new therapeutic targets in undifferentiated endometrial sarcoma. Gynecol. Obstet. Investig..

[8] Takai N., Desmond J.C., Kumagai T., Gui D., Said J.W., Whittaker S., Miyakawa I., Koeffler H.P. (2004). Histone deacetylase inhibitors have a profound antigrowth activity in endometrial cancer cells. Clin. Cancer Res..

[9] Chen Y., Tsai Y.H., Tseng S.H. (2017). HDAC inhibitors and RECK modulate endoplasmic reticulum stress in tumor cells. Int. J. Mol. Sci..

[10] Wong C.C., Qian Y., Yu J. (2017). Interplay between epigenetics and metabolism in oncogenesis: Mechanisms and therapeutic approaches. Oncogene.

[11] Li L.H., Zhang P.R., Cai P.Y., Li Z.C. (2016). Histone deacetylase inhibitor, Romidepsin (FK228) inhibits endometrial cancer cell growth through augmentation of p53–p21 pathway. Biomed. Pharmacother..

[12] Gonfloni S., Iannizzotto V., Maiani E., Bellusci G., Ciccone S., Diederich M. (2014). P53 and Sirt1: Routes of metabolism and genome stability. Biochem. Pharmacol..

[13] Lim C.S. (2007). Human SIRT1: A potential biomarker for tumorigenesis?. Cell Biol. Int..

[14] Moore R.L., Dai Y., Faller D.V. (2012). Sirtuin 1 (SIRT1) and steroid hormone receptor activity in cancer. J. Endocrinol..

[15] Moore R.L., Faller D.V. (2013). SIRT1 represses estrogen-signaling, ligand-independent ERα-mediated transcription, and cell proliferation in estrogen-responsive breast cells. J. Endocrinol..

[16] Park E.Y., Woo Y., Kim S.J., Kim D.H., Lee E.K., De U., Kim K.S., Lee J., Jung J.H., Ha K.T. (2016). Anticancer effects of a new SIRT inhibitor, MHY2256, against human breast cancer MCF-7 Cells via regulation of MDM2-p53 binding. Int. J. Biol. Sci..

[17] Yang Q., Wang B., Gao W., Huang S., Liu Z., Li W., Jia J. (2013). SIRT1 is downregulated in gastric cancer and leads to G1-phase arrest via NF-κB/Cyclin D1 signaling. Mol. Cancer Res..

[18] Peck B., Chen C.Y., Ho K.K., Di Fruscia P., Myatt S.S., Coombes R.C., Fuchter M.J., Hsiao C.D., Lam E.W. (2010). SIRT inhibitors induce cell death and p53 acetylation through targeting both SIRT1 and SIRT2. Mol. Cancer Ther..

[19] Hoffmann G., Breitenbücher F., Schuler M., Ehrenhofer-Murray A.E. (2014). A novel sirtuin 2 (SIRT2) inhibitor with p53-dependent pro-apoptotic activity in non-small cell lung cancer. J. Biol. Chem..

[20] Wang J., Kim T.H., Ahn M.Y., Lee J., Jung J.H., Choi W.S., Lee B.M., Yoon K.S., Yoon S., Kim H.S. (2012). Sirtinol, a class III HDAC inhibitor, induces apoptotic and autophagic cell death in MCF-7 human breast cancer cells. Int. J. Oncol..

[21] Eckschlager T., Plch J., Stiborova M., Hrabeta J. (2017). Histone deacetylase inhibitors as anticancer drugs. Int. J. Mol. Sci..

[22] Lara E., Mai A., Calvanese V., Altucci L., Lopez-Nieva P., Martinez-Chantar M.L., Varela-Rey M., Rotili D., Nebbioso A., Ropero S. (2009). Salermide, a Sirtuin inhibitor with a strong cancer-specific proapoptotic effect. Oncogene.

[23] Duan H., Heckman C.A., Boxer L.M. (2005). Histone deacetylase inhibitors down-regulate *bcl-2* expression and induce apoptosis in t(14;18) lymphomas. Mol. Cell. Biol..

[24] Kwon S.H., Ahn S.H., Kim Y.K., Bae G.U., Yoon J.W., Hong S., Lee H.Y., Lee Y.W., Lee H.W., Han J.W. (2002). Apicidin, a histone deacetylase inhibitor, induces apoptosis and Fas/Fas ligand expression in human acute promyelocytic leukemia cells. J. Biol. Chem..

[25] Librizzi M., Spencer J., Luparello C. (2016). Biological effect of a hybrid anticancer agent based on kinase and histone deacetylase inhibitors on triple-negative (MDA-MB231) breast cancer cells. Int. J. Mol. Sci..

[26] Chen D., Xu M., Wu B., Chen L. (2017). Histone deacetylases in hearing loss: Current perspectives for therapy. J. Otol..

[27] Lee Y.J., Won A.J., Lee J., Jung J.H., Yoon S., Lee B.M., Kim H.S. (2012). Molecular mechanism of SAHA on regulation of autophagic cell death in tamoxifen-resistant MCF-7 breast cancer cells. Int. J. Med. Sci..

[28] Ahn M.Y., Jung J.H., Na Y.J., Kim H.S. (2008). A natural histone deacetylase inhibitor, Psammaplin A, induces cell cycle arrest and apoptosis in human endometrial cancer cells. Gynecol. Oncol..

[29] Ahn M.Y., Chung H.Y., Choi W.S., Lee B.M., Yoon S., Kim H.S. (2010). Anti-tumor effect of apicidin on Ishikawa human endometrial cancer cells both in vitro and in vivo by blocking histone deacetylase 3 and 4. Int. J. Oncol..

[30] Lin P.-C., Hsieh H.-Y., Chu P.-C., Chen C.S. (2018). Therapeutic opportunities of targeting histone deacetylase isoforms to eradicate cancer stem cells. Int. J. Mol. Sci..

[31] Bartosch C., Monteiro-Reis S., Almeida-Rios D., Vieira R., Castro A., Moutinho M., Rodrigues M., Graça I., Lopes J.M., Jerónimo C. (2016). Assessing sirtuin expression in endometrial carcinoma and non-neoplastic endometrium. Oncotarget.

[32] Li M., Luo J., Brooks C.L., Gu W. (2002). Acetylation of p53 inhibits its ubiquitination by Mdm2. J. Biol. Chem..

